# Economic (gross cost) analysis of systematically implementing a programme of advance care planning in three Irish nursing homes

**DOI:** 10.1186/s13104-016-2048-9

**Published:** 2016-04-26

**Authors:** Ronan O’Sullivan, Aileen Murphy, Rónán O’Caoimh, Nicola Cornally, Anton Svendrovski, Brian Daly, Carol Fizgerald, Cillian Twomey, Ciara McGlade, D. William Molloy

**Affiliations:** Centre for Gerontology and Rehabilitation, University College Cork, St Finbarrs Hospital, Cork City, Ireland; School of Economics, University College Cork, College Road, Cork City, Ireland; Health Research Board, Clinical Research Facility Galway, National University of Ireland, Galway, Geata an Eolais, University Road, Galway City, Ireland; COLLAGE (COLLaboration on AGEing), University College Cork, Cork City, Ireland; Catherine McAuley School of Nursing and Midwifery, University College Cork, Cork, Ireland; UZIK Consulting Inc., 86 Gerrard St E, Unit 12D, Toronto, ON M5B 2J1 Canada; College of Medicine and Health, Brookfield Health Sciences Complex, University College Cork, College Road, Cork City, Ireland

**Keywords:** Advanced care planning, Advanced care directives, Economic analysis

## Abstract

**Background:**

Although advance care planning (ACP) and the use of advanced care directives (ACD) and end-of-life care plans are associated with a reduction in inappropriate hospitalisation, there is little evidence supporting the economic benefits of such programmes. We assessed the economic impact (gross savings) of the Let Me Decide (LMD) ACP programme in Ireland, specifically the impact on hospitalisations, bed days and location of resident deaths, before and after systematic implementation of the LMD-ACP combined with a palliative care education programme.

**Methods:**

The LMD-ACP was introduced into three long-term care (LTC) facilities in Southern Ireland and outcomes were compared pre and post implementation. In addition, 90 staff were trained in a palliative care educational programme. Economic analysis including probabilistic sensitivity analysis was performed.

**Results:**

The uptake of an ACD or end-of-life care post-implementation rose from 25 to 76 %. Post implementation, there were statistically significant decreases in hospitalisation rates from baseline (hospitalisation incidents declined from 27.8 to 14.6 %, *z* = 3.96, *p* < 0.001; inpatient hospital days reduced from 0.54 to 0.36 %, *z* = 8.85, *p* < 0.001). The percentage of hospital deaths also decreased from 22.9 to 8.4 %, *z* = 3.22, *p* = 0.001. However, length of stay (LOS) increased marginally (7–9 days). Economic analysis suggested a cost-reduction related to reduced hospitalisations ranging between €10 and €17.8 million/annum and reduction in ambulance transfers, estimated at €0.4 million/annum if these results were extrapolated nationally. When unit costs and LOS estimates were varied in scenario analyses, the expected cost reduction owing to reduced hospitalisations, ranged from €17.7 to €42.4 million nationally.

**Conclusions:**

Implementation of the LMD-ACP (ACD/end-of-life care plans combined with palliative care education) programme resulted in reduced rates of hospitalisation. Despite an increase in LOS, likely reflecting more complex care needs of admitted residents, gross costs were reduced and scenario analysis projected large annual savings if these results were extrapolated to the wider LTC population in Ireland.

## Background

By 2050, the Irish population aged over 65 years will double to 1.4 million. Currently, 5 % reside in long term care (LTC) but while the proportion may remain unchanged, the total number of residents is expected to rise [1]. Increases will be even greater in those over 85 years, of whom 21 % currently reside in LTC [2]. Over the next 10 years, approximately 1000 extra LTC beds will be required, each year, to accommodate the rising need for LTC [3]. Increasing numbers of people will die in LTC [4–6] making it essential that LTC facilities provide the highest quality end-of-life care.

Patient involvement in medical decision-making is encouraged and advance care planning (ACP) allows people to consider their wishes for end-of life care and to state their wishes should they become incapable of communicating them later. ACP is defined as a continuous communication and decision-making process between patients, families and healthcare professionals, addressing issues relating to end-of-life care prior to the patient requiring such care. ACP may result in the development of a written document called an advance care directive (ACD), a frequent but facultative result of the ACP process, or an end-of-life care plan. An ACD is a record of an informed decision, valid only if a competent individual makes it voluntarily and is used or acted upon only if the person becomes incompetent to make medical decisions. Depending on the jurisdiction, an ACD can be legally binding. An end-of-life care plan is created between individuals who lack capacity, their family, and healthcare professionals to plan for future healthcare decisions. It is not legally binding, rather a road map to guide the decision making process. While a resident who lacks decision-making capacity is ineligible to complete an ACD, any expressed views in relation to end-of-life care can be documented in this end-of-life care plan. ACP including the creation of either an ACD or end-of-life care plan offers a unique opportunity to optimise care, promote autonomy, empower patients and maximise resource use [7]. It promotes collaborative care, reduces heath inequalities by increasing access to palliative care, improves satisfaction with end-of-life care and facilitates choice of place of death [8–11].

Ireland is on the cusp of major changes in relation to advance decision-making. The Law Reform Commission made strong recommendations to give ACDs a legal basis and the new Assisted Decision-Making (capacity) Bill 2013, once enacted, will provide a statutory framework for ACDs. Before ACDs become widely available in Ireland, we need to understand the clinical value and impact they will have, including their cost effectiveness and feasibility. There is poor evidence to support the use of ACP interventions including ACDs and end-of-life care plans for older people, particularly those with cognitive impairment [12–14]. The paucity of supporting evidence is due to a lack of quasi-experimental, controlled before-after or randomised controlled trials (RCT) particularly in LTC [7]. Most research in this area is descriptive or qualitative often focusing on ACD or end-of-life care plan completion rates, rather than on their effects on quality of end-of-life care or on their economic impact [15–17].

Let Me Decide (LMD) is an established ACP programme, originally developed in Canada [7] (LMD-ACP) and is a selected good practice initiative within the Collaboration on Ageing (COLLAGE), Ireland’s 3 Star Reference Site for the European Innovation Partnership on Active and Healthy Ageing (http://www.collage-ireland.eu/initiatives/specific-action-group-members/let-me-decide/) [18, 19]. LMD was systematically implemented in pilot studies [20–22] and in a RCT in LTC in Canada [8]. The majority of residents completing directives chose to remain in the LTC facility and receive appropriate palliative end-of-life care [8]. Similar results were found in the United States with significant cost savings in their final week of life [23]. Higher costs were associated with worse quality of death [23]. In Australia, 21 nursing homes and two hospitals using LMD were compared to a geographically separate hospital and thirteen nursing homes. During a 3-year follow up period there was a significant reduction in emergency ambulance calls in the intervention homes (p < 0.0019) and a 25 % reduction in hospital bed occupancy by intervention home residents compared to control homes (relative risk 0.74; p < 0.0001) [9].

The current study evaluated the feasibility of systematically implementing the LMD-ACP programme in three Irish LTC facilities together with a palliative care workshop. The introduction of this programme into these pilot LTC settings in Ireland has been mostly a positive experience and the programme was well received [24]. This paper describes the economic gross cost analysis of a pilot study, assessing the impact on hospitalisations, location of residents’ deaths and number of days the nursing home residents spent in acute hospital care, before and after systematic implementation.

## Methods

Three LTC facilities were recruited from the south of Ireland. These included two private and one publically funded (community nursing unit) nursing homes, totaling 290 beds at baseline. All residents, aged ≥65 years, in participating units were eligible for inclusion in the programme. Residents were recruited throughout the study period. New residents were included in the analysis such that bed occupancy was maximized throughout follow-up. This number grew from baseline to the beginning of the post-implementation phase as the bed capacity of two of the units increased during this period. Beds occupied by participants excluded from the study (i.e. those residents <65 years) did not contribute to the analysis of outcomes, which were calculated as average annual event rates per available occupied bed across the three units. Statistical and economic analysis is expanded upon below. The study adhered to the tenets of the Declaration of Helsinki (1975). The study was approved by the Clinical Research Ethics Committee of the Cork Teaching Hospitals and residents provided informed consent where possible. Assent was obtained for those who lacked capacity.

### The advance care planning and palliative care education intervention

Nursing staff from participating homes completed two half-day workshops on the LMD-ACP programme focusing on the ethical, legal and practical considerations of ACP with residents and their families in LTC. A separate education programme in palliative care was delivered over another two half-days. The first palliative care half-day, attended by nurses and healthcare assistants, focused on the palliative care approach, communication at end-of-life and issues relating to grief and bereavement. The second half-day, delivered to nursing staff only, focused on symptom assessment and management. This education aimed to provide staff with the skills to deliver holistic, patient-centered care, using the principals of palliative care and the ability to recognise when timely referral to specialist palliative care services would benefit dying residents, to ensure they received high quality end-of-life care.

Each study home was given a detailed implementation manual that included a policy on completing ACDs/ACPs, decision aids for engaging residents, documentation templates, structured forms and educational resources for residents and families. Live ACP demonstrations with a sample of residents and families in front of small groups of nursing staff in all three study sites were given. Senior nursing staff were offered support in monthly feedback meetings to discuss issues arising during the implementation process. Residents were approached in turn, on the unit for existing residents or on admission to the unit for new residents, during the set-up phase. Residents and families who expressed an interest were provided with information about the LMD-ACP programme by senior nurses. In addition, one-off, evening and weekend, information sessions for families and residents were delivered in the homes by senior members of the research team. After, residents and families were asked if they wished to participate, resident’s capacity to complete the LMD-ACD was then assessed [25]. Each competent resident who voluntarily decided to engage in the LMD-ACP process was given a verbal and written explanation of the study. Residents were asked to sign a consent form and were assured that any information collected would be treated as strictly confidential. They were also informed that they were free to withdraw their consent to participate at any time, that they were under no obligation to complete an ACD, that their decision to engage in the ACP process would not affect the quality or amount of healthcare that they would receive, and that there would be no risks involved by their participation in the study. Those residents who completed an ACD were informed that they could change or withdraw their ACD at any time they wished. Likewise, families of incompetent residents who voluntarily decided to engage in the LMD-ACP process were invited to complete an end-of-life care plan. They were then also asked to sign consent and were given the same assurances as competent residents. An opportunity for the resident to provide a personal statement is fostered and included in the documentation.

### Implementation of ‘Let Me Decide’

The LMD-ACP implementation is divided into four steps [26]:

1. The first step in the LMD-ACP process screens cognition, using the Standardised Mini-Mental State Examination (SMMSE) [27]. This determines whether a resident is likely to have sufficient capacity to engage and understand the ACP process. Residents who were deemed suitable (SMMSE score >10) were offered the opportunity to engage in the LMD-ACP process.
2. Education is provided with trained senior nurses informing residents and family members individually that a new advance directive program is being implemented and that they have an opportunity to be educated about directives and to complete one. The nurses explain the five sections of the LMD directive, the terminology such as the type of care they would want if their condition was “reversible/acceptable” and the type of care they would want if their condition was “irreversible/intolerable.” Practical examples are provided.
3. Competency is then assessed. Each resident’s capacity to complete the LMD-ACD was assessed using the Screening Instrument to Assess Capacity to Complete an Advanced Directive [25, 28]. The expected high prevalence of cognitive impairment in the LTC population [29] underlined the need to include this assessment of resident capacity as a critical step in the completion of a valid ACD. When residents lacked capacity to complete ACD’s, their end-of-life care choices were discussed. Where possible, the resident was included in this discussion together with their family (with the residents permission). In some instances the resident was unable through severe impairment of cognition to participate. In these circumstances their families were invited to discuss end-of-life care choices, taking consideration of any previously expressed wishes of the resident or with knowledge of the resident and their values, what they felt the resident would have wanted. Encapsulating the decisions discussed, and with the agreement of those involved, an end-of-life care plan was completed by the resident’s doctor and nurse.
4. Once the resident is deemed competent, choices are reviewed and documented on the directive. The directive is then signed by the patient, substitute decision makers (usually family), and physician.

### Economic analysis

An economic (gross costs) analysis was performed to estimate the economic impact of reduced hospitalisations anticipated with the implementation of the LMD-ACP. This cost analysis employed standard techniques that required the identification, measurement and valuation of resources as per Drummond et al. [30]. Resources identified were inpatient hospitalisations (by episode and length of admission) and ambulance transfers. To measure resources used, results were employed to estimate the probability of hospitalisations among nursing home residents prior to and post implementation of LMD-ACP. Length of stay (LOS) was estimated and each hospitalisation was associated with an ambulance transfer. With regards to valuing the resources employed, secondary data were employed to estimate the average cost per episode [31], average per diem cost [31] and cost of ambulance transfer [32]. An average per diem cost was estimated to account for the variation between national average LOS associated with the inpatient casemix cost and the LOS indicated in this study.

Scenario analyses were used to investigate the effect of changes to LOS and gross cost per diem. Firstly, LOS is varied using admission evidence from a hospital admissions database (a large, university teaching hospital and level one trauma centre, covering southern Ireland and serving a population of more than 1,173,000 people), used as a reference point to provide LOS data on transfers from nursing homes for the same time periods as the LMD-ACP study. Secondly, the per diem rate estimated in the baseline analysis, which may be considered conservative relative to estimates employed in other studies. For example, the Strategy for Long Term Care in Ireland estimated the cost of acute care to be €6000/week, which would be €857/day, based on BDO International auditors’ data [33]. The per diem gross cost was also varied to determine the effect on the results if a higher cost was used. Finally, a probabilistic sensitivity analysis was performed as a means of addressing usual uncertainties surrounding parameters like those employed in this study. This required characterising uncertainty in input parameters and propagating uncertainty through the model using a Monte Carlo simulation. The results of this present the implications of parameter uncertainty [34].

### Statistical analysis

Descriptive statistics were used to summarise hospital days and death counts in the three LTC units for each project phase: baseline (January 2010–June 2012), and post-implementation (July 2013–June 2015). The implementation phase (between these two phases) during set-up of the study was examined separately and is not included in this analysis as this was the time when education was provided and staff were becoming familiar with implementing the LMD-ACP process, i.e. it was a education phase. In addition to counts, four ratios (proportions) for each project phase were calculated:

- Annual death rate (number of deaths per year ÷ average number of residents across all three nursing units).
- Percentage of deaths in hospital (number of hospital deaths ÷ total number of deaths).
- Hospitalisation rate based on incidents of hospitalisation (number of hospitalisations per year ÷ average number of residents across all three nursing units).
- Hospitalisation rate based on inpatient hospital days (hospital bed days ÷ number of resident days).

For the purpose of analysis, we compared rates in the post intervention phase with the baseline (pre intervention) phase. As the study was designed to explore a decline in each of the three rates in the post-intervention phase, a series of one-sided z-tests for independent samples proportions was conducted comparing rates in the baseline phase with the post-intervention phase.

## Results

The number of deaths, the number of days in acute hospital care (hospital bed days per resident) and the ratios for each phase of the project in all three pilot nursing homes are provided in Table 1. Two of the homes added new beds during the study necessitating the use of percentages and rates in the analyses because the absolute number of residents changed during the study period. At baseline 290 residents were available and this increased to 304 at the beginning of the post implementation phase. All residents were aged over 65 years. Their characteristics did not change significantly between the pre and post-implementation phases. The mean age was 85.9 years. The majority were female (67.3 %) and the most had cognitive impairment (mean Abbreviated Mental Test Score 3.3/10). Of those that died, more than three quarters of respondents (76.5 %) were with a relative. Only three residents across all study sites were excluded as they were aged <65 years. The participation rate of those who received information varied. Prior to the study, 25 % had some form of end-of-life care plan in place. This increased to 76 % post implementation, ranging from 57 to 90 % across the three sites (12 % of these were ACDs completed by the residents). Only 10 % of residents were deemed to have had capacity to complete their own ACD. In all, 84 % of those who died during the post-implementation period had an end-of-life care plan in place, compared to 89 % of residents who died in the nursing homes, while 50 % of those who died following transfer to acute care had one in place. In total, four residents who had an end-of-life care plan died in an acute hospital; three of these stated that these residents would decline hospital transfer.

**Table 1:** Hospitalisations, length of stay (LOS) and deaths across the three long-term care units including comparison with reference site

	Pre-implementation	Post-implementation
Months	30	24
Number of beds (excluding those declining to participate)	287	301
Deaths per year (average)	87	83
Annual death rate (average)	30.3 %	27.6 %
Deaths in Hospital (average per year)	8	7
Percentage of deaths in hospital (average)	22.9. %	8.4 %
Total deaths	218	166
Hospitalisations per year	80	44
Hospitalisation rate (based on hospitalisation incidents)	27.9 %	14.6 %
Average LOS per stay	7.02	9.07
Average LOS for same period in reference hospital site amongst those transferred from nursing homes	9.89	8.58
Hospital bed days (per month)	1403 (46.8)	798 (33.3)
Hospitalisation rate (based on hospital days)	0.54 %	0.36 %

Combining data from the three nursing homes revealed a decrease in the annual mortality rate, from 30.3 % at baseline to 27.6 % in the post-implementation phase, which was not statistically significant z = 0.74, p = 0.23. There was a significant decline in the proportion of deaths in hospital, from 22.9 % at baseline to 8.4 % post-implementation, z = 3.22, p = 0.001. There was also a significant reduction, by almost half, in hospitalisation rates. Hospitalisation, derived from number of incidents, decreased from 27.9 to 14.6 % between baseline and post-implementation, z = 3.93, p < 0.001; hospitalisation calculated by the number of hospital days declined from 0.54 to 0.36 % from baseline to post-implementation, z = 8.85, p < 0.001. These results are shown in Table 2.

**Table 2:** Statistical comparison of outcomes (average annual rate) between pre and post implementation phases

	Pre-implementation (%)	Post-implementation (%)	One-sided z-test
Annual death rate (average)	30.3	27.6	*z* = 0.74, *p* = 0.23
Percentage of deaths in hospital (average per year)	22.9	8.4	*z* = 3.22, p = 0.001
Hospitalisation rate (average per year based on hospitalization incidents)	27.8	14.6	*z* = 3.96, *p* < 0.001
Hospitalisation rate (average per year based on hospital days)	0.54	0.36	*z* = 8.85, *p* < 0.001

### Economic cost of all hospitalisations

The direct gross costs associated with hospitalisations of nursing home residents include inpatient costs and ambulance transfer costs. At baseline (January 2010 to June 2012), the probability of hospitalisations per resident was 0.28 per annum on average. This reduced to 0.15 post implementation of the LMD-ACP process (July 2013–June 2015) on average per annum. The average LOS of these hospitalisations however, increased from seven at baseline (January 2010–June 2012) to nine post implementation of the LMD-ACP process (July 2013–June 2015).

Reasons for admission included pneumonia, chronic obstructive pulmonary disease, urosepsis, stroke, cardiac failure and bowel obstruction. The associated diagnosis related groups (DRGs) were collected from the Ready Reckoner [31] and using the inpatient casemix cost per case, an average cost per episode was estimated at €4081 (standard deviation €3328). Averaging the costs per DRG across the national average LOS, yielded an average daily cost of €491 (standard deviation €59). The cost of ambulance transfers, sourced from Gannon et al. and adjusted for inflation is estimated to be €97/transfer [32].

Nursing Home Ireland indicates the population of nursing home residents to be 33,000. Extrapolating the results of the LMD-ACP study to the nursing home population in Ireland, the expected change in hospitalisation costs owing to reduced hospitalisations can be estimated. Expected hospitalisations pre-implementation of LMD-ACP for this population is estimated to be 9186 annually. Applying the average cost per episode (€4081) to this yields an average cost of €37.5 million per annum (gross). If the reduced probability of hospitalisations of 0.15 were to persist, hospitalisations amongst nursing home residents would be reduced to 4824 per annum. Applying the average cost per episode to this yields an average cost of €19.7 million per annum, indicating a cost reduction of €17.8 million (gross). In addition, ambulance transfers would be reduced by 4362, yielding a further cost reduction of €423,453 per annum (gross). Alternatively, if the cost of inpatient hospitalisations was estimated using the daily average cost (€491/day) using LOS results from this study, the expected cost reduction per annum is a more conservative €10.2 million per annum (gross). This reflects that the LOS amongst nursing home residents is less than the national average per DRG. These results are summarised in Table 3.

**Table 3:** Economic cost analysis: comparison pre and post implementation of the Let Me Decide Advanced Care Planning (LMD-ACP) programme (gross costings)

Unit of analysis	Parameter	Pre implementation (Jan 2010–Jun 2012)	Post implementation (July 2013–Jun 2015)	Difference
	Probability of Hospitalisation per resident^a^	0.28	0.15	–
	Total Hospitalisation Episodes Nationally^b^	9186	4824	4362
	Length of Stay LMD-ACP	7.02	9.07	2.05
a. Episode of care	€4081/Episode^c^	€37,487,265	€19,686,419	€17,800,847
b. Length of stay	€491/day DRG^d^	€31,630,876	€21,472,704	€10,158,173
Ambulance transfers	€97/transfer^e^	€891,761	€468,308	€423,453

^a^Based on data from this LMD-ACP study

^b^Based on 33,000 nursing home beds nationally

^c^Based on average inpatient case mix cost across common diagnosis related groups (DRGs)

^d^Daily average from inpatient case mix cost across common DRGs

^e^Gannon et al. [32]

### Scenario analysis

The sensitivity of the analysis was tested by varying two parameters in a scenario analyses: LOS and per diem cost. Firstly, the baseline analysis above employed LOS evidence from this study, which indicated an increase in average LOS between baseline (January 2010–June 2012) and post implementation of LMD-ACP (July 2013–June 2015). However, evidence from the university hospital reference sites’ database reveals that for the same periods: baseline (January 2010–June 2012) and post implementation of the LMD-ACP process (July 2013–June 2015), the average LOS for residents admitted from nursing homes decreased from 9.89 to 8.58 days. Adjusting for these LOS data increases the expected cost reduction to €24.3 million (gross) (Scenario analysis 1).

Secondly, in the absence of a national cost reference database, it is not a surprise that hospitalisation costs vary between analyses. The per diem rate estimated in the baseline analysis above (€491) is conservative relative to estimates employed in other studies. For example, the Strategy for Long Term Care in Ireland estimated the cost of acute care to be €6000/week, which would be €857/day [33]. Applying this higher cost per diem to the LOS estimates from this study indicates an expected cost reduction of €17.7 million (gross) (Scenario analysis 2).

A third scenario analysis considers the effect of employing LOS data from the reference hospital and the average per diem cost of €857/day. Here the expected cost reduction, owing to reduced hospitalisations, is €42.4 million (gross) (Scenario analysis 3). These are summarised in Table 4.

**Table 4:** Scenario analysis: comparison pre and post implementation of the Let Me Decide Advanced Care Planning (LMD-ACP) programme (gross costings)

Scenario	Pre implementation (January 2010–June 2012)	Post implementation (July 2013–June 2015)	Difference
1. LOS—reference hospital data^a^ and €491/day^c^	€44,594,350	€20,304,890	€24,289,461
2. LOS—LMD-ACD data^b^ and €857/day^d^	€55,232,926	€37,495,017	€17,737,909
3. LOS—reference hospital data and €857/day^d^	€77,869,371	€35,455,814	€42,413,557

^a^Based on reference hospital length of stay (LOS) data

^b^Based on LMD-ACD LOS data

^c^Daily average from inpatient case mix cost across common diagnosis related groups (DRGs)

^d^BDO International auditors’ data [33]

### Probabilistic sensitivity analysis

To account for parameter uncertainty a probabilistic sensitivity analysis (PSA) was performed. The results including upper and lower percentiles at the 95 % confidence level for the baseline cost analyses and the scenarios are summarised on Table 5 and show that for the baseline analysis and scenario analyses implementation of the LMD-ACP programme continues to yield an average cost reduction owing to reduced hospitalisations. However, where LOS from the pilot study is used, the PSA identifies some uncertainty surrounding the cost reduction. Nevertheless, the results of the PSA indicate that there is an 89 % probability that costs will be reduced and 11 % probability that the increased LOS increases costs. However, when episode of care or LOS from the reference hospital database is used to estimate inpatient costs, there is a 100 % probability that costs are reduced.

**Table 5:** Results from probabilistic sensitivity analysis: average costs and upper and lower percentiles (gross costings)

Analysis	Pre implementation (January 2010–June 2012) average € millions (lower and upper percentiles 95 %)	Post implementation (July 2013–June 2015) average € millions (lower and upper percentiles 95 %)	Difference average € millions (lower and upper percentiles 95 %)
a. Baseline: €4081/episode of hospitalisation^c^	37.82 (2.65–119.34)	19.87 (1.37–64.10)	17.95 (1.15–58.90)
b. Baseline: LMD-ACP LOS^a^ and €491/day^d^	32.49 (18.96–52.34)	21.83 (12.46–35.40)	10.67 (−6.10–30.69)
Ambulance transfers^e^	0.89 (0.55–1.34)	0.47 (0.27–0.73)	0.42 (0.19–0.73)
*Scenario analyses*			
1. LOS—reference hospital data^b^ and €491/day^f^	44.69 (25.84–70.51)	20.30 (13.75–28.31)	24.39 (6.05–48.55)
2. LOS—LMD-ACD Data^a^ and €857/day^f^	56.71 (34.61–87.58)	38.11 (22.48–60.20)	18.60 (−10.87–52.14)
3. LOS—reference hospital data^b^ and €857/day^f^	77.98 (47.24–118.78)	35.43 (25.59–47.01)	42.55 (10.72–83.16)

^a^Based on reference hospital length of stay (LOS) data

^b^Based on LMD-ACP LOS data

^c^€4081—based on average inpatient case mix cost across common diagnosis related groups (DRGs)

^d^€491/day—daily average from inpatient case mix cost across common DRGs

^e^€97/transfer Gannon et al. [32]

^f^€857/day—BDO International auditors’ data [33]

## Discussion

This paper presents the economic cost impact of the systematic implementation of the LMD-ACP programme in three LTC facilities in the south of Ireland. Hospitalisations and deaths were measured for two and half years before (baseline i.e. pre-implementation) and two years after the programme was implemented (post-implementation phase). The results show that there was a significant reduction in hospital transfers and number of days residents spent in hospital following the implementation of the programme, comparing the two phases. There was also a reduction of in-hospital deaths, although this did not reach statistical significance. The results of this study enable us to estimate the gross cost burden associated with the decision to transfer LTC residents to hospital. Inpatient admissions and ambulance transfers were quantified directly and invasive interventions indirectly, amongst those who had in-hospital deaths. Given the anticipated reductions in hospitalisations, the expected cost reductions ranged between €10 and €17.8 million per annum (gross) with an additional €0.4 million for a reduction in ambulance transfers, depending on which costing methodology is used. The rationale for using unit costs per episode, which results in projected savings of approximately €17.8 million per year (gross), if the LMD-ACP programme were introduced nationwide in Ireland, lies in the planned shift towards activity based costing envisaged for the Irish healthcare system, whereby costs will be based on episodes of care. However, at present until activity based costing becomes a reality in Ireland, LOS as a unit of analysis is a more conservative and possibly realistic estimate. The results of the PSA reaffirm the cost savings.

The three scenarios presented in the scenario analysis all result in large savings due to a projected reduction in hospitalisations. This is despite an increased LOS, which likely reflects an increased casemix associated with more complex inpatient management for those who required hospital transfer. Thus, even though LOS increased, the number of bed days used overall reduced.

Given the lack of a reference cost database, it is difficult to calculate the exact cost of a bed day in Ireland—to account for this uncertainty, a range is provided: the conservative estimate of €491 per day and the more expensive €857 per day rate based upon DRGs (see Table 3). Irrespective of which cost estimate per day is used, the savings are substantial. With regards to the use of LOS estimates, the increased LOS in the trial, we found that this wasn’t in line with the local reference hospital (a university hospital tertiary referral centre) trends during the period, so the scenario analysis considers cost if the number of hospitalisations were to reduce without impacting LOS. The two baseline analyses and the scenario analysis all suggest the same conclusion, although to varying amounts depending on the LOS and rates used, with the potential cost savings likely to be between €10 and €42 million annually (gross).

The findings of this study are similar to other studies looking at end-of-life planning. In the United States, end-of-life discussions alone, without using a specific ACP programme, resulted in a 35 % reduction in costs in the last week of life, albeit for patients with advanced cancer [23]. In Singapore, ACP as part of a programme to improve end-of-life care for nursing home residents found a per-resident cost savings of SGD$7129 (confidence interval: SGD$4544–SGD$9714) over the last 3 months of life [17].

Based upon a previous qualitative assessment, the LMD-ACP and palliative care education programmes were well received by the staff of these units [24] and residents and their families [25]. Following implementation, the uptake of some form of ACP (ACDs or end-of-life care plans) in the three homes increased to 90 % of residents. In this study, 10 % of residents had capacity to complete their own ACD. For those who lacked capacity, end-of-life care plans were completed by the medical team, following discussions with the family and with the resident where possible. This is compatible with current Irish law. Nursing staff reported that, in general, families were very keen to be involved in the end-of-life care planning process and that families of residents who lacked capacity to complete an ACD or ACP consistently asked for low levels of intervention for their relative at the end-of-life. The majority requested that the resident should not be transferred to acute care hospitals, and be kept in the nursing home at the end-of-life, if possible. Following implementation of the programme, feedback from staff indicated that a lack of time to deliver ACP was one of the biggest challenges they encountered, particularly for those with cognitive impairment, and that protected time would be helpful to deliver ACP effectively in an unhurried manner [24].

This study has a number of limitations that provide reasons to be cautious about the estimates provided. The greatest limitation is that it was a before-after intervention study with the LTC units acting as their own historical controls (only the time period varied) and that baseline and end-point demographic data were not routinely available. This may have introduced bias in that the characteristics of patients including their health status may have changed, likely deteriorating over time. That said, this would favour the null and serve to potentially strengthen the data supporting a reduction in hospitalisations and length of stay. This could also create potential bias in that the effects seen might reflect other changes in hospital admission and healthcare policy locally, and on a national level in Ireland. To gain some historical controls other than these homes themselves we reviewed hospitalisation rates into the regions tertiary referral centre, as a reference site, and found that there was no significant change in hospitalisations from LTC facilities during this period. If anything, there was a trend towards an increase in hospitalisations from LTC facilities. This may reflect system changes such as alterations to data collection or coding, potentially limiting the results of this study. The scenario analysis presented here including aggregated level data is theoretical to consider the possible gross savings if the preliminary results presented were confirmed. Thus, to confirm the cost saving estimates presented here, a RCT is required. Further, this paper presents the results from different costing approaches, which may have over or under-estimated savings. Choosing which costing methods to employ is challenging and any inference made, where the marginal effect of explanatory variables is assessed, is substantially influenced by the costing method [35]. Different models were presented in this paper although others may have more accurately reflected potential cost savings. In addition, no data were available on the cost of implementation limiting this study to a gross cost analysis rather than a full evaluation. Finally, the question of appropriateness of hospitalisation wasn’t addressed in this study. While it is possible that this intervention may have resulted in inappropriate decisions to withhold transfer, as this study was conducted rigorously as a clinical trial and supervised by a steering committee, which examined each decision to transfer afterwards, it is unlikely that residents care was unduly affected. Following implementation of the programme a decision to transfer out was made in advance and in the majority of cases competent patients or families of incompetent patients requested that the person should stay in the nursing home and not be transferred. In this case patients remained unless they could not be kept comfortable in the unit. In the event of an unexpected event, each decision to transfer was made by the nursing staff and the primary care physician supervising the unit in conjunction with the resident and or the family members on a case-by-case basis with reference to the patients stated preferences. On the other hand, prior to this study when a resident became acutely ill, the decision to transfer was made by a primary care physician. Prior to implementation 25 % had either an end-of-life care plan or an ACD, which in the majority of cases was a ‘do not attempt resuscitation’ order and did not address transfer. In these cases the default position was the transfer of the patient. Several studies have examined the proportion of hospital admissions of nursing home residents that were inappropriate or avoidable [36, 37]. A study in the United Kingdom looking at the cost saving represented by potentially avoidable admissions (35 of 483 admissions), by caring for such patients in alternative locations found it would have saved approximately £5.9 million per year for the two hospitals involved with the study [38].

## Conclusions

In summary, this before-after trial suggests that there was a significant reduction in hospitalisation rates following the systematic implementation of the LMD-ACP and palliative care education programme in three LTC facilities in southern Ireland. It shows that there were significant cost savings associated with this reduction in admissions. Despite an increase in average LOS, likely reflecting more complex care needs of admitted residents, costs were estimated to be reduced and scenario analysis projected large significant annual cost savings associated with this reduction in admissions. The economic cost analysis indicates that should the reduced hospitalisations amongst LTC residents as a result of the LMD-ACP process be transferrable to the general LTC population in Ireland then it has the potential to substantially reduce inpatient and ambulance transfer costs. Such cost reductions were consistent when costing per diem or per episode and using a range of costs. This expands and strengthens findings from two other RCTs in Canada and Australia and supports the generalisability of these findings. A randomized trial of this programme in now underway in Ireland to confirm the findings of this pilot study.

## Abbreviations

ACD: advanced care directives
ACP: advance care planning
DRGs: diagnosis related groups
LOS: length of stay
LMD: Let Me Decide
LTC: long-term care
PSA: probabilistic sensitivity analysis
RCT: randomised controlled trials
SGD: Singapore dollar
SMMSE: Standardised Mini-Mental State Examination

## References

[1] http://www.cardi.ie/userfiles/Long%20Term%20Care%20(Web).pdf. Last Accessed 24 Nov 2015.

[2] Central Statistics Office (2008). Population and labour force projections 2011–2041.

[3] Layte R, editor. Projecting the impact of demographic change on the demand for and delivery of Healthcare in Ireland, ESRI Research Series No. 13. Dublin: Economic and Social Research Institute; 2009.

[4] Ahmad S, O’Mahony MS (2005). Where older people die: a retrospective population-based study. QJM.

[5] Cartwright A (1991). Changes in life and care in the year before death 1969–1987. J Public Health Med.

[6] Hunt R, Maddocks I. Terminal care in South Australia: historical aspects and equity issues. In: Clark D, Hockley J, Ahmedzai S, editors. Facing death—new themes in palliative care. Buckingham: Open University Press; 1997. p. 101–5.

[7] Weathers E, Cornally N, Coffey A, Daly E, O’Caoimh R, Mollow DW. Planning for end-of-life: a systematic review of randomised controlled trials conducted in older adults. Maturitas; 2016, under review. 10.1016/j.maturitas.2016.06.016 27451328

[8] Molloy DW, Guyatt GH, Russo R, Goeree R, O’Brien BJ, Bédard M (2000). Systematic implementation of an advance directive program in nursing homes: a randomized controlled trial. JAMA.

[9] Caplan (2006). Advance care planning and hospital in the nursing home. Age Aging.

[10] Brinkman-Stoppelenbeurg A, Rietjens JAC, Van Der Heide A (2014). The effects of advance care planning on end-of-life care: a systematic review. Palliat Med.

[11] Houben CHM, Spruit MA, Groenen MTJ, Wouters EFM, Janssen DJA (2014). Efficacy of advance care planning: a systematic review and meta-analysis. J Am Med Dir Assoc.

[12] Robinson L, Dickinson C, Rousseau N, Beyer F, Clark A, Hughes J (2012). A systematic review of the effectiveness of advance care planning interventions for people with cognitive impairment and dementia. Age Ageing.

[13] Hockley J, Watson J, Oxenham D, Murray SA (2010). The integrated implementation of two end-of-life care tools in nursing care homes in the UK: an in-depth evaluation. Palliat Med.

[14] McClelland B, Ashton S, Roe B, Mazhindu D, Gandy R (2008). End of life care: an evaluation of the implementation of the gold standards framework and the liverpool care pathway for people with dementia on five care settings across Greater Manchester.

[15] Durbin CR, Fish AF, Bachman JA, Smith KV (2010). Systematic review of educational interventions for improving advance directive completion. J Nurs Scholarsh.

[16] Tamayo-Velázquez MI, Simón-Lorda P, Villegas-Portero R, Higueras-Callejón C, García-Gutiérrez JF, Martínez-Pecino F, Barrio-Cantalejo IM (2010). Interventions to promote the use of advance directives: an overview of systematic reviews. Patient Educ Couns.

[17] Teo WSK, Raj AG, Tan WS, Ng CWL, Heng BH, Leong IYO (2014). Economic impact analysis of an end-of-life programme for nursing home residents. Palliat Med.

[18] Sweeney C, Molloy DW, O’Caoimh R, Bond R, Hynes H, McGlade C, Shorten G (2013). European innovation partnership on active and healthy ageing: Ireland and the COLLAGE experience. Ir J Med Sci.

[19] O’Caoimh R, Sweeney C, Hynes H, McGlade C, Cornally N, Daly E (2015). COLLaboration on AGEing-COLLAGE: Ireland’s three star reference site for the European Innovation Partnership on Active and Healthy Ageing (EIP on AHA). Eur Geriatr Med.

[20] Molloy DW, Guyatt G (1991). A comprehensive health care directive in a home for the aged. Can Med Assoc J.

[21] Molloy DW, Urbanyi M, Horsman J, Guyatt GH, Bédard M (1992). Two years’ experience with a comprehensive health care directive in a Home for the Aged. Ann RCPSC.

[22] Molloy DW, Guyatt G, Goeree R, Jubelius R, Urbanyi M, Flett N, Bedard M, King D (1997). A comprehensive health-care directive for competent and incompetent residents of a home for the aged. Ann RCPSC.

[23] Zhang B, Wright AA, Huskamp HA, Nilsson ME, Maciejewski ML, Earle CC (2009). Health care costs in the last week of life: associations with end-of-life conversations. Arch Intern Med.

[24] Cornally N, McGlade C, Weathers E, Daly E, Fitzgerald C, O’Caoimh R (2015). Evaluating the systematic implementation of the ‘Let Me Decide’ advance care planning programme in long term care through focus groups: a user’s perspective. BMC Palliat Care.

[25] Molloy DW, Silberfeld M, Darzins P, Guyatt GH, Singer PA, Rush B (1996). Measuring capacity to complete an advance directive. J Am Geriatr Soc.

[26] Molloy DW, Russo R, Stiller A, O’Donnell MJ (2000). How to implement the “Let Me Decide” advance health and personal care directive program. JCOM.

[27] Molloy DW, Alemayehu E, Roberts R (1991). Reliability of a standardized mini-mental state examination compared with the traditional mini-mental state examination. Am J Psychiatry.

[28] Molloy DW, Russo R, Pedlar D, Bédard M (2000). Implementation of advance directives among community-dwelling veterans. The Gerontologist.

[29] Cahill S, Diaz-Ponce AM, Coen RF, Walsh C (2010). The underdetection of cognitive impairment in nursing homes in the Dublin area. The need for on-going cognitive assessment. Age Ageing.

[30] Drummond M, Sculpher M, Claxton K, Stoddart G, Torrance G. Methods for the economic evaluation of health care programmes. 4th ed. Oxford: Oxford University Press; 2015.

[31] National Casemix Programme. Ready Reckoner 2013. Ready Reckoner of Acute Hospital inpatient and daycase activity and costs (summarised by DRG) relating to 2011 costs and activities. HSE; 2013.

[32] Gannon B, O’Shea E, Hudson E. Technical report 1. The economic costs of falls and fractures in people aged 65 and over in Ireland. Technical report to NCAOP/HSE/DOHC. Irish Centre for Social Gerontology, National University of Ireland Galway; 2007.

[33] BDO. Health’s aging crisis: time for action. A future strategy for Ireland’s long-term residential care sector. 2014. http://www.bdo.ie/sites/default/files/BDO%20Report%20Health’s%20Ageing%20Crisis%20-%20Time%20for%20Action%20February%202014.pdf Last Accessed 25 Nov 2015.

[34] Briggs AH, Claxton K, Sculpher MJ (2006). Decision modelling for health economic evaluation.

[35] Geue C, Lewsey J, Lorgelly P, Govan L, Hart C, Briggs A (2012). Spoilt for choice: implications of using alternative methods of costing hospital episode statistics. Health Econ.

[36] Finucane P, Wundke R, Whitehead C, Williamson L, Baggoley C (2000). Use of in-patient hospital beds by people living in residential care. Gerontology.

[37] Ouslander JG, Lamb G, Perloe M, Givens JH, Kluge L, Rutland T (2010). Potentially avoidable hospitalizations of nursing home residents: frequency, causes, and costs. J Am Geriatr Soc.

[38] Gardiner C, Ward S, Gott M, Ingleton C (2014). Economic impact of hospitalisations among patients in the last year of life: an observational study. Palliat Med.

